# International Journal of Health Policy and Management (IJHPM): A Decade of Advancing Knowledge and Influencing Global Health Policy (2013-2023)

**DOI:** 10.34172/ijhpm.2023.8124

**Published:** 2023-05-24

**Authors:** Mina Moradzadeh, Mohammad Karamouzian, Sahar Najafizadeh, Vahid Yazdi-Feyzabadi, Ali-Akbar Haghdoost

**Affiliations:** ^1^Health Services Management Research Center, Institute for Futures Studies in Health, Kerman University of Medical Sciences, Kerman, Iran; ^2^Centre On Drug Policy Evaluation, St. Michael’s Hospital, Toronto, ON, Canada; ^3^Dalla Lana School of Public Health, University of Toronto, Toronto, ON, Canada; ^4^HIV/STI Surveillance Research Center, and WHO Collaborating Center for HIV, Kerman University of Medical Sciences, Kerman, Iran; ^5^Modeling in Health Research Center, Institute for Futures Studies in Health, Kerman University of Medical Sciences, Kerman, Iran

## Introduction

 The *International Journal of Health Policy and Management *(IJHPM) is a peer-reviewed, gold open access, and scientific multidisciplinary journal, with a specific focus on health policy and management research. It serves as a platform that brings together specialists from various fields, including health policy, health management, health economics, epidemiology, public health, global health, and social and public policy into a dynamic academic environment. The journal aims to foster effective communication among policy-makers, health system researchers, decision-makers, legislators, practitioners, educators, administrators, students, and allied health professionals, facilitating collaboration and knowledge sharing within the research and healthcare delivery systems.

 The IJHPM was founded by Dr. Akram Khayatzadeh-Mahani (Former Assistant Professor of Health Policy at Kerman University of Medical Sciences. She is currently an assistant professor at the University of Regina) in early 2013. Its first issue was published on June 25, 2013, marking its entry into the competitive international landscape. Approaching its 10th anniversary in June 2023, IJHPM has achieved notable milestones. Over this period, IJHPM published 12 volumes and 112 issues, including four special issues^[1]^. From 2013 to 2023, there has been a consistent growth in the number of monthly manuscripts submitted to IJHPM, with an average of 16 submissions in 2013 to 71 in 2023. Out of a total of 7107 submissions received, 1698 peer-reviewed articles were published in IJHPM, representing approximately 24% of the total submissions^[2]^.

 The journal’s papers are indexed in several abstracting and indexing databases, and they are highly regarded by scientists. IJHPM is currently indexed in various prestigious databases, including the Web of Science Journal Citation Reports (WoS JCR) (Social Sciences Edition), WoS Science Citation Index Expanded (SciSearch^®^), WoS Journal Citation Reports (Science Edition), WoS Social Sciences Citation Index^®^, WoS Current Contents^®^ (Social and Behavioral Sciences), MEDLINE, Scopus, EBSCOhost, PubMed Central (PMC), and Directory of Open Access Journals (DOAJ). In June 2022, the WoS JCR released its last impact factor (IF) of 4.967, positioning IJHPM as the 10th-ranked journal out of 88 in the “Health Policy & Services” category. This achievement places IJHPM in the first quartile (Q1).

 IJHPM was free of charge for eight years, from June 2013 to June 2021. However, in order to cover the expenses associated with editorial tasks, administrative support, technical tasks, and indexing services, we transitioned from the Green Open Access publishing model to the Gold Open Access. To facilitate publishing, we have taken into account discounts and waivers for authors from low- and middle-income countries (LMICs), students, and IJHPM reviewers.

## An Overview of IJHPM’s Advances

###  Indexing and Abstracting Databases

 In the beginning, the five-year vision was to be indexed in PMC, Medline, Scopus, and WoS by 2018. Therefore, we designed the journal’s processes and workflow, such as the peer review process, technical standards, and the website, based on the four abstracting and indexing evaluation criteria and also followed international and reputable guidelines like the International Committee for Medical Journal Editors (ICMJE), Committee on Publication Ethics (COPE), and World Association of Medical Editors (WAME). Furthermore, the IJHPM’s editorial office continually evaluates the quality of processes and implemented some innovative actions to advance its rank among leading scientific journals. For instance, in order to standardize sex and gender reporting in research publications, we began incorporating the Sex and Gender in Research (SAGER) guideline^1^ into our author guide in May 2021 and encouraged our authors to follow it. In April 2023, we switched to the CRediT format in accordance with the updated technical requirements in PubMed for reporting the authors’ contributions.

 The first goal of IJHPM was achieved remarkably quickly, within six months, as the journal gained visibility in PMC in 2014. The second goal was to secure indexing in MEDLINE, the largest and oldest biomedical database in the world,^2^ with more complex inclusion criteria compared to PMC, Scopus, and even WoS.^3^ To accomplish this, we followed specific policies to highlight the distinctive nature of our publication and set IJHPM apart from other journals in the same field. We established the knowledge translation line of IJHPM, which comprises two sections: ‘Key Messages’ and ‘Video Summary.’ In the ‘Key Messages’ section, authors of all original articles are requested to present the key findings of their research under two separate headings namely: ‘Implications for Policy Makers’ and ‘Implications for Public.’ Additionally, we introduced ‘Video Summary’ as another IJHPM knowledge translation activity. This initiative involves inviting authors of selected publications to prepare video summaries of their articles, which are then published on the IJHPM YouTube Channel. Currently, there are 157 videocasts and podcasts available on this channel. Simultaneously, we commenced efforts to attract new editorial board members, reviewers, and authors from around the world, with a particular emphasis on engaging individuals from resource-limited settings and LMICs. In 2015, our collective efforts paid off, and IJHPM was indexed in Medline as a young journal.

 To accomplish our next set of goals, which included indexing in WoS and Scopus, we maintained a continuous effort to monitor and adhere to the evaluation criteria of these two databases. Additionally, we closely monitored the citation status of our journal in Google Scholar. Recognizing that citation metrics play a crucial role in the evaluation process of WoS and Scopus,^4,5^ we implemented new strategies to enhance the visibility and promotion of our published papers. In pursuit of this objective, we utilized various academic social networks, such as Academia, ResearchGate, LinkedIn, and Mendeley^[3]^ to promote our published papers. Furthermore, IJHPM actively engaged with the wider public through popular social networks such as Facebook and Twitter, ensuring that all papers were promptly shared on these platforms following their online publication. Moreover, we encouraged authors to actively disseminate their papers among their own academic networks, expanding the reach and impact of our publications.

**Figure 1:**
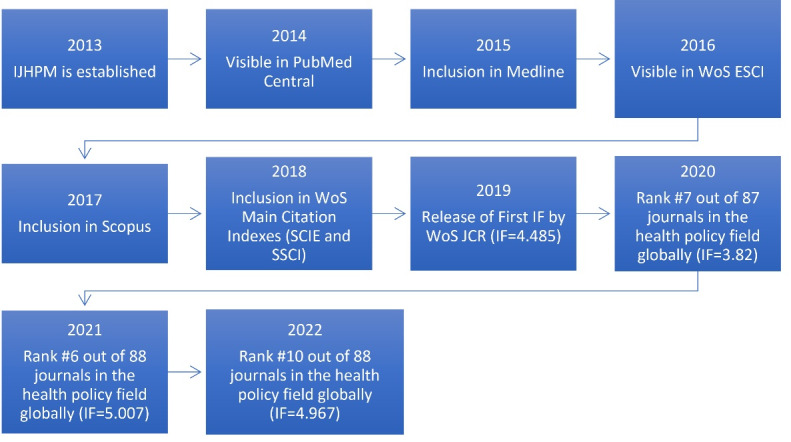
IJHPM Timeline in Abstracting and Indexing Databases. Abbreviations: ESCI, Emerging Sources Citation Index; JCR, Journal Citation Reports; IF, impact factor; WoS, Web of Science; SCIE, Science Citation Index Expanded; SSCI, Social Sciences Citation Index; IF, impact factor.

 In 2016, the journal gained visibility in WoS Emerging Sources Citation Index (ESCI), marking a notable achievement. Subsequently, by 2017, IJHPM was accepted for inclusion in Scopus, further solidifying its position in the academic community. One of the primary objectives outlined in IJHPM’s five-year vision was to attain an official IF. This goal was successfully realized by the end of 2018, with the first IF of IJHPM being released by JCR 2019, garnering an impressive IF of 4.485. This achievement underscored the growing influence and impact of IJHPM within its field. The progress of IJHPM’s citation database has continued to thrive, culminating in its current ranking of 10th position in the field, as reported by JCR 2021. The persistent growth and recognition of IJHPM’s scholarly contributions are evident in its citation database progress. A graphical representation of the timeline progress of IJHPM is presented in Figure 1, offering a visual demonstration of the journal’s remarkable trajectory.

###  IJHPM Editorial Board Members and Staff

 IJHPM has successfully attracted the collaboration of renowned scholars from around the world, who have established themselves as leading experts in their respective fields. Currently, our editorial board consists of 24 esteemed members, comprising eight women and 16 men, representing 12 countries across five continents. All members actively contribute to reviewing papers, writing editorial papers, and the exchange of ideas aimed at enhancing the overall quality of the journal. Over the past decade, these dedicated board members have played an instrumental role in publishing 69 editorial papers.^6-83^ Furthermore, IJHPM has published four special issue editorials,^9,12,35,64^ three guest editorials, and two joint global editorials on the climate change movement.^7,8^

 Over the past decade, IJHPM has experienced several modifications in its editorial board and office members. However, the most notable change occurred in September 2021 when Dr. Akram Khayatzadeh-Mahani resigned from her position as the Editor-in-Chief after dedicating eight years (January 2013 to September 2021) to establishing and enhancing IJHPM’s reputation. Although her absence is deeply felt, IJHPM is grateful to have her ongoing support as the founding editor (October 2021-current).

 In October 2021, Professor Ali-Akbar Haghdoost, the former director-in-charge of IJHPM, assumed the position of Editor-in-Chief at IJHPM. Alongside him, three new associate editors joined the IJHPM team to assist with daily editorial duties and work closely with the editorial office members. Currently, IJHPM has a team of five in-house editors, six staff members, and one consultant. The detailed information of editorial board members, in-house editors, and staff is presented in Table S1 (Supplementary file 1).

###  IJHPM Peer-Review and Publication Processes

**Figure 2:**
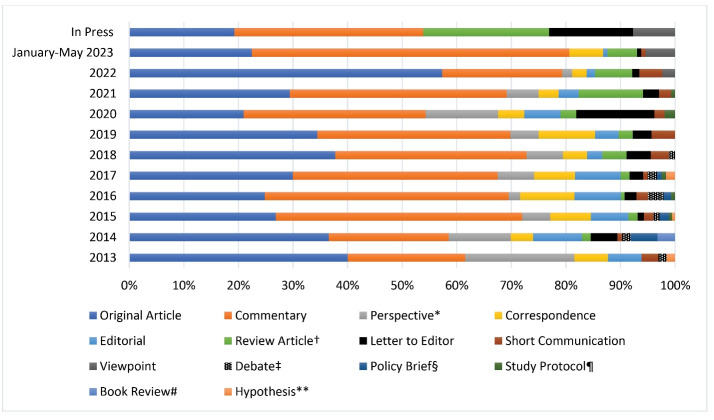
IJHPM Article Types and Frequencies. * Since 2023, we have stopped publishing ‘Perspective’ articles.
^†^ Review articles consist of Systematic Review, Meta-Analysis, Systematic Reviews and Meta-Analysis, Meta-Synthesis, and Scoping Reviews.
^‡^ Since 2019, we have stopped publishing ‘Debate’ articles.
^§^ Since 2018, we have stopped publishing ‘Policy Brief’ articles.
^¶^ Since 2021, we have stopped publishing ‘Study Protocol’ articles.
^#^ Since 2015, we have stopped publishing ‘Book Review’ articles.

 Over the course of 10 years, IJHPM has contributed significantly to academic discourse by publishing a total of 1698 peer-reviewed articles. Throughout its publication history, IJHPM has featured a diverse range of scientific article types, with a total of 14 distinct categories. However, over time, six of these article types have been discontinued, resulting in the current selection of eight types that are presently published: editorial, viewpoint, review articles, original article, short communication, commentary, correspondence, and letter to editor. For a comprehensive overview of the distribution of each article type published by IJHPM over the past decade, refer to Figure 2.

**Figure 3:**
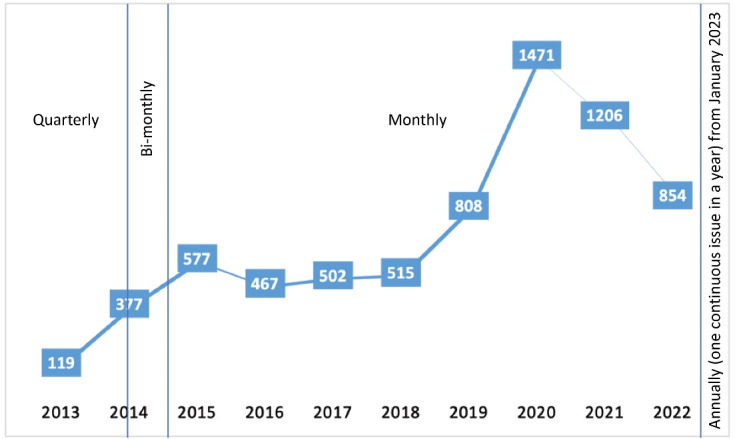
IJHPM Submission Trends From June 2013 to December 2022.

 On average, IJHPM receives approximately 690 manuscript submissions annually with a 9% acceptance rate. Over the years, there has been a noticeable upward trend in the number of submissions, particularly since 2018 when the submission rate accelerated even further. Notably, the highest number of submissions was recorded in 2020, with a total of 1471 manuscripts received. This upward trend in submissions could potentially be attributed to the journal’s commendable IF score and increased visibility within the academic community. However, since 2021, there has been a decrease in the number of submissions, which may be linked to changes in the journal’s business model, including the implementation of article processing charges (APC) for authors. The submission trend of IJHPM is shown in Figure 3.

 To ensure the high quality of published articles, the editor-in-chief and section editors of IJHPM conduct a thorough pre-screening of all submissions. This pre-screening process involves assessing the manuscripts for their methodological rigor, originality, contribution to knowledge and practice, as well as the use of English language. Based on the outcome of this pre-screening, manuscripts will be either fast rejected, sent back to authors for revision and resubmit, or sent for external peer review. Manuscripts that meet the IJHPM’s priority and quality criteria will undergo a double-blind peer-review process to evaluate the content quality. In the pre-screening stage, approximately 80% of IJHPM submissions are rejected, and only 20% proceed to the external peer review. This stringent selection process guarantees that only the highest quality and most relevant articles are considered for publication in IJHPM.

 The IJHPM upholds a rigorous double-blind peer-review process that prioritizes speed, fairness, and the publication of high-quality articles. Reviewers, selected based on their scientific background, expertise, and authors’ recommendations, are required to disclose conflicts of interest and maintain strict confidentiality. The IJHPM strives to obtain at least two strong and comprehensive reviews for each manuscript. The Editor-in-Chief and section editors carefully consider reviewers’ comments and prepare decision letters, which undergo multiple rounds of review before final acceptance or rejection. Once accepted, the technical team will publish the author-accepted version of the manuscript (AAM) while producing the PDF proof. Publishing the AAM version expedites the dissemination of accepted manuscripts, making them available on the IJHPM website within three days of acceptance.

 The journal currently follows an annual publication schedule, with any changes in publication frequency are shown in Figure 1. This meticulous process, combined with the commitment to transparency and prompt dissemination, ensures that IJHPM maintains its reputation as a premier platform for scholarly discourse in health policy and management.

###  Diversity of IJHPM Authors and Reviewers

 The IJHPM has published articles from 5794 authors across 105 countries. The majority of authors are affiliated with the United Kingdom (n = 651), the United States (n = 649), Iran (n = 621), Canada (n = 590), and Australia (n = 543). Table 1 highlights the top 10 most productive authors.

**Table 1:** IJHPMs’ Top 10 Productive Authors

	**Name**	**Affiliation**	**Country**	**Number of Articles**	**References**
1	Mohammad Karamouzian	University of Toronto	Canada	17	^50,84-99^
2	Russell Mannion	University of Birmingham	UK	14	^54,56-59,77,100-107^
3	Ali-Akbar Haghdoost	Kerman University of Medical Sciences	Iran	14	^40,50,62,86,88,90-93,108-112^
4	Ronald Labonté	University of Ottawa	Canada	14	^9,52,53,79,113-122^
5	Martin McKee	London School of Hygiene and Tropical Medicine	UK	14	^61,79,123-134^
6	Patrick P. T Jeurissen	Radboud University Medical Center	The Netherlands	12	^135-146^
7	Leon Bijlmakers	Radboud University Medical Center	The Netherlands	12	^10,34,147-156^
8	Ruairí Brugha	Royal College of Surgeons in Ireland	Ireland	12	^19,20,34,79,148,150,152,153,156-159^
9	Fran Baum	Flinders University	Australia	11	^119,122,160-168^
10	Rob Baltussen	Radboud University Medical Center	The Netherlands	11	^10,11,147,154,155,169-174^

 Since its inception in July 2013, the journal has established a dynamic platform that fosters global collaboration and facilitates discussions on contemporary issues through its commentary and correspondence line. This initiative invites scholars to contribute commentaries following the publication of various article types, such as original research, reviews, and editorials. The commentaries received are then compiled and shared with the main authors, who provide responses that are subsequently published as correspondence papers. These collections of commentaries and correspondence papers create a special compendium, enabling in-depth exploration of specific topics with contributions from scholars worldwide. From July 2013 to May 2023, a total of 136 collections have been published, encompassing 589 commentaries and 93 correspondence papers.

 Since its establishment, the IJHPM has garnered the collaboration of 4339 reviewers from 108 countries. Notable, the majority of reviewers are from Australia (n = 280), Canada (n = 272), the United States (n = 260), the United Kingdom (n = 159), and China (n = 151). The selection process for reviewers employed by IJHPM is characterized by its unique approach, which involves comprehensive database searches to identify individuals with expertise relevant to the manuscripts under consideration. Invitations to serve as reviewers are then sent via the journal’s management system, with the goal of securing at least five agreements for research manuscripts and three agreements for short manuscripts. To achieve this goal, up to 50 reviewers may be invited per manuscript. The IJHPM’s global reach is reflected in the diverse backgrounds of its reviewers, as highlighted in Table 2, which showcases the top 20 reviewers.

**Table 2:** IJHPM’s Top 20 Reviewers

	**Name**	**Affiliation**	**Country**	**Number of Reviewed Manuscripts**	**Average Time to Review (Days)**
1	Aidin Aryankhesal ^orcid^	University of East Anglia	United Kingdom	35	14
2	Mohammad Reza Baneshi ^orcid^	University of Queensland	Australia	28	1
3	Martin McKee ^orcid^	London School of Hygiene and Tropical Medicine	United Kingdom	25	20
4	Ghobad Moradi ^orcid^	Kurdistan University of Medical Sciences	Iran	23	28
5	Mohammad Hajizadeh ^orcid^	Dalhousie University	Canada	20	30
6	Joel Lexchin ^orcid^	York University	Canada	17	1
7	Ehsan Zarei ^orcid^	Shahid Beheshti University of Medical Sciences	Iran	17	4
8	Mohammad Karamouzian ^orcid^	University of Toronto	Canada	13	27
9	Ronald Labonté ^orcid^	University of Ottawa	Canada	12	4
10	Helen Schneider ^orcid^	University of the Western Cape	South Africa	12	20
11	Krishna Hort ^orcid^	University of Melbourne	Australia	11	19
12	Phillip Baker ^orcid^	Deakin University	Australia	10	26
13	Philip Dalinjong ^orcid^	University of Technology Sydney	Ghana	10	34
14	Matthew Fisher ^orcid^	University of Adelaide	Australia	10	16
15	Gill Harvey ^orcid^	Flinders University	Australia	10	39
16	Simon Rushton ^orcid^	University of Sheffield	United Kingdom	10	21
17	Edwine Barasa ^orcid^	KEMRI-Wellcome Programme	Kenya	9	50
18	Carlos Bruen ^orcid^	Royal College of Surgeons	Ireland	9	8
19	Unni Gopinathan ^orcid^	Norwegian Institute of Public Health	Norway	9	49
20	Dimitri Renmans ^orcid^	University of Antwerp	Belgium	9	23

###  IJHPM Status in Citation Databases

 Based on data from the WoS database, the IJHPM has received a notable total of 12 210 citations. The JHPM H-index in WoS is 41, and the average number of citations per article is 8.3. In the “Health Policy & Services” (SSCI) category, the IJHPM holds an impressive 10th position out of 88 journals, and in the “Health Care Sciences & Services” (SCIE) category, it ranks 20th out of 109 journals. Furthermore, the IJHPM has achieved a Q1 ranking in both categories, highlighting its prominence and impact in the field. The journal reached its highest IF of 5.007 based on the JCR 2020. Table 3 provides a comprehensive overview of the IJHPM’s IF trend and ranking data in WoS.

**Table 3:** IJHPM Impact Factor Trend and Ranking Data in Web of Science

	**2-Year IF**	**5-Year IF**	**JCI**^a^	**Health Policy & Services (Quartile)**	**Health Care Sciences & Services (Quartile)**	**Cited Half-life**^b^ ** (y)**	**Citing Half-life**^c^ ** (y)**	**Immediacy Index**^d^
JCR 2021	4.967	5.418	0.99	ranks 10 among 88 journals (Q1)	ranks 20 among 109 journals (Q1)	3.6	6.4	1.787
JCR 2020	5.007	4.909	1.15	ranks 6 among 88 journals (Q1)	ranks 13 among 107 journals (Q1)	3.6	6	4.533
JCR 2019	3.821	NA	1.16	ranks 7 among 88 journals (Q1)	ranks 14 among 102 journals (Q1)	3.4	6.3	1.472
JCR 2018	4.485	NA	0.91	ranks 6 among 82 journals (Q1)	ranks 12 among 98 journals (Q1)	2.8	8.4	1.66

Abbreviations: JCR, Journal Citation Reports; IF, impact factor; NA, not available; Q1, quartile 1. ^a^The Journal Citation Indicator (JCI) is the average Category Normalized Citation Impact (CNCI) of citable items (articles & reviews) published by a journal over a recent three-year period. The average JCI in a category is 1. Journals with a JCI of 1.5 have 50% more citation impact than the average in that category. It may be used alongside other metrics to help you evaluate journals. ^b^The Cited Half-Life is the median age of the items in this journal that were cited in the JCR year. Half of a journal’s cited items were published more recently than the cited half-life. ^c^The Citing Half-Life is the median age of items in other publications cited by this journal in the JCR year. ^d^The Immediacy Index is the count of citations in the current year to the journal that references content in this same year. Journals that have a consistently high Immediacy Index attract citations rapidly.

**Figure 4:**
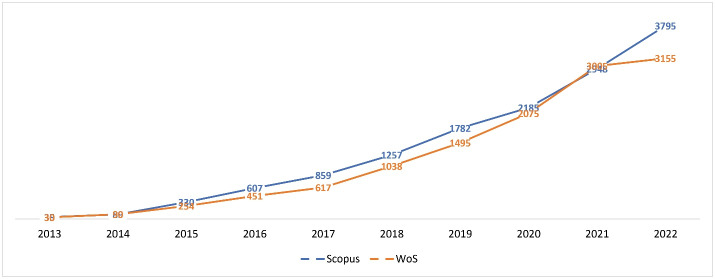
IJHPM Citation Trend in Web of Science and Scopus.

 From 2013, the IJHPM amassed a significant total of 13 877 citations in Scopus. The IJHPM H-index in Scopus is 45, and the journal’s current CiteScore is 6.5, securing the top position among 36 journals in the “leadership and management” category. The IJHPM is listed in five subject categories in Scopus and is classified as a Q1 journal in all of them. Table 4 presents a detailed record of the IJHPM’s metrics in Scopus over time. Figure 4 shows the upward trend in citation rates for the IJHPM in both WoS and Scopus.

**Table 4:** IJHPM CiteScore trend and Ranking Data in Scopus

	**CiteScore**	**Leadership and Management (Quartile)**	**Health Information Management (Quartile)**	**Health Policy (Quartile)**	**Health (Social Science) (Quartile)**	**Management, Monitoring, Policy and Law (Quartile)**
2021	6.5	ranks 1 among 36 journals (Q1)	ranks 10 among 39 journals (Q1)	ranks 20 among 265 journals (Q1)	ranks 19 among 323 journals (Q1)	ranks 54 among 376 journals (Q1)
2020	5	ranks 1 among 29 journals (Q1)	ranks 8 among 39 journals (Q1)	ranks 26 among 242 journals (Q1)	ranks 24 among 293 journals (Q1)	ranks 68 among 355 journals (Q1)
2019	3.5	ranks 3 among 31 journals (Q1)	ranks 10 among 33 journals (Q2)	ranks 55 among 239 journals (Q2)	ranks 46 among 275 journals (Q1)	ranks 99 among 333 journals (Q2)
2018	3.1	ranks 4 among 30 journals (Q2)	ranks 12 among 27 journals (Q2)	ranks 70 among 229 journals (Q2)	ranks 58 among 262 journals (Q2)	ranks 106 among 313 journals (Q2)
2017	3.2	NA	NA	ranks 57 among 226 journals (Q2)	NA	NA
2016	2.3	NA	NA	ranks 91 among 216 journals (Q2)	NA	NA

Abbreviations: NA, not available; Q1, quartile 1.

Table 5 highlights the top 10 most frequently cited articles published by the IJHPM in the past decade. Notably, the article titled “The development of a critical appraisal tool for use in systematic reviews addressing questions of prevalence”^175^ authored by Zachary Munn, Sandeep Moola, Dagmara Riitano, and Karolina Lisy in 2014 has received the highest number of citations overall is.

**Table 5:** IJHPM’s Top 10 Highly Cited Articles

**Article Title**	**Authors**	**Document Type**	Times Cited*	**DOI**
The development of a critical appraisal tool for use in systematic reviews addressing questions of prevalence	Munn, Zachary; Moola, Sandeep; Riitano, Dagmara; Lisy, Karolina	Original Article	637	10.15171/ijhpm.2014.71
Factors influencing healthcare service quality	Mosadeghrad, Ali Mohammad	Original Article	216	10.15171/ijhpm.2014.65
Unequal Gain of equal resources across racial groups	Assari, Shervin	Perspective	173	10.15171/ijhpm.2017.90
What is resilience and how can it be nurtured? a systematic review of empirical literature on organizational resilience	Barasa, Edwine; Mbau, Rahab; Gilson, Lucy	Review Article	170	10.15171/ijhpm.2018.06
Collaboration and co-production of knowledge in healthcare: opportunities and challenges	Rycroft-Malone, Jo; Burton, Christopher R.; Bucknall, Tracey; Graham, Ian D.; Hutchinson, Alison M.; Stacey, Dawn	Editorial	123	10.15171/ijhpm.2016.08
Governance and capacity to manage resilience of health systems: towards a new conceptual framework	Blanchet, Karl; Nam, Sara L.; Ramalingam, Ben; Pozo-Martin, Francisco	Perspective	121	10.15171/ijhpm.2017.36
Defining integrated knowledge translation and moving forward: a response to recent commentaries	Kothari, Anita; McCutcheon, Chris; Graham, Ian D.	Correspondence	112	10.15171/ijhpm.2017.15
Knowledge, moral claims and the exercise of power in global health	Shiffman, Jeremy	Editorial	112	10.15171/ijhpm.2014.120
Occupational stress and turnover intention: implications for nursing management	Mosadeghrad, Ali Mohammad	Original Article	104	10.15171/ijhpm.2013.30
Out-of-pocket payments, catastrophic health expenditure and poverty among households in Nigeria 2010	Aregbeshola, Bolaji Samson; Khan, Samina Mohsin	Original Article	96	10.15171/ijhpm.2018.19

* This data was extracted from Web of Science on May 2023.

###  IJHPM Hosted an International Seminar

 On April 19, 2014, the IJHPM team held an impactful international seminar focused on the theme of “improving quality and efficiency of health services by introducing market incentives, competition, and choice: opportunities and challenges” (Supplementary file 2). The seminar featured an esteemed keynote speaker, Marianna Fotaki who provided valuable insights and perspectives on the main theme of the seminar, and Dr. Alex Scott-Samuel, Professor Carol Molinari, Dr. Owen Adams, and Professor Ian Greener contributed to the seminar by sending pre-recorded videos. The event also witnessed the active participation of renowned Iranian scholars in the field, who attended in person and engaged in fruitful discussions, further enriching the seminar’s outcomes.

## Conclusion

 This editorial aimed to provide an assessment of the progress and evolution of the IJHPM over the past decade. Despite being a relatively new journal in the health policy and management field based in a resource-limited LMIC, the IJHPM has demonstrated remarkable growth in both quality and quantity measures. With its emphasis on timely high-quality publication and a commitment to fostering scholarly discourse, the IJHPM has become one of the leading and reputable journals for researchers in the health policy and management field. By implementing a culture that values quality, respect, fairness, inclusiveness, teamwork and collaboration, innovation, and speed, the journal has established itself as a valuable resource for academics, policymakers, and practitioners. These values permeate all IJHPM activities and we will keep them in focus while continuing to expand our capacity to meet the growing demands for high-quality publications. Moving forward, the IJHPM is poised to continue its upward trajectory and contribute to the advancement of health policy and management research. These achievements would have been impossible without the continuous and hard efforts of highly dedicated and committed IJHPM editorial office members, active and supportive editorial board members, dedicated reviewers, respected authors, and supportive loyal readers.

## Acknowledgment

 We thank the IJHPM office staff, particularly Hafez Mohammadhassanzadeh, Fatemeh Molaei, and Zeinab Golestanikhah, for their great assistance.

## Ethical issues

 Not applicable.

## Competing interests

 All authors are in-house editors at the IJHPM.

## Endnotes


^[1]^ Special Issue on Analysing the Politics of Health Policy Change in LMICs, July 2021; edited by Lucy Gilson, Zubin Cyrus Shroff, and Maylene Shung-King. Special Issue on WHO-CHOICE Update, November 2021; edited by Melanie Y. Bertram, Tessa Tan Torres Edejer, and led by Mohammad Hajizadeh. Special Issue on Political Economy of Food Systems, December 2021; edited by Phillip Baker, Jennifer Lacy-Nichols, Ronald Labonté, and Owain Williams. Special Issue on CHS-Connect, January 2022; edited by Helen Schneider, Anna-Karin Hurtig, Joseph Zulu, Uta Lehmann, Miguel San Sebastian, and Charles Michelo
^[2]^ We have included here the invited-only articles (i.e., editorial, commentary, and correspondence articles). Without the invited-only articles, the acceptance rate is 9% [output date: May 21, 2023].
^[3]^ On March 2021, Mendeley retired the public groups so the public page of IJHPM in Mendeley were removed (https://blog.mendeley.com/2020/11/02/weve-listened-to-our-users-and-are-refocusing-on-whats-important-to-them/); last access: May 21, 2023

## Supplementary files


Supplementary file 1. IJHPM Editors Including in-house Editors, Editorial Board Members, and Staff Members.

Supplementary file 2. IJHPM International Seminar’s Agenda.
