# Supplementary material for: International Journal of Health Policy and Management (IJHPM): A Decade of Advancing Knowledge and Influencing Global Health Policy (2013-2023)
Source: Int J Health Policy Manag. 2023 May 24;12:8124. doi: 10.34172/ijhpm.2023.8124 (PMC10425691; doi:10.34172/ijhpm.2023.8124)
Supplement: Supplementary file 1 — IJHPM Editors Including in-house Editors, Editorial Board Members, and Staff Members. [file ijhpm-12-8124-s001.pdf]

https://ijhpm.com
Int J Health Policy Manag 2023;12:8124

IJHPM
International Journal of Health Policy and Management

Supplementary file 1

Article title: International Journal of Health Policy and Management (IJHPM): A Decade of Advancing Knowledge and Influencing Global Health Policy (2013-2023)
Journal name: International Journal of Health Policy and Management (IJHPM)

Authors' information: Mina Moradzadeh[1*], Mohammad Karamouzian[2,3,4], Sahar Najafizadeh[1], Vahid Yazdi-Feyzabadi[1], Ali-Akbar Haghdoost[5]
[1]Health Services Management Research Center, Institute for Futures Studies in Health, Kerman University of Medical Sciences, Kerman, Iran
[2]Centre On Drug Policy Evaluation, St. Michael's Hospital, Toronto, ON, Canada.
[3]Dalla Lana School of Public Health, University of Toronto, Toronto, ON, Canada.
[4]HIV/STI Surveillance Research Center, and WHO Collaborating Center for HIV, Kerman University of Medical Sciences, Kerman, Iran.
[5]Modeling in Health Research Center, Institute for Futures Studies in Health, Kerman University of Medical Sciences, Kerman, Iran.
*Correspondence to: Mina Moradzadeh, Email: minamoradzadeh.medlis@gmail.com

Citation: Moradzadeh M, Karamouzian M, Najafizadeh S, Yazdi-Feyzabadi V, Haghdoost AA. International Journal of Health Policy and Management (IJHPM): a decade of advancing knowledge and influencing global health policy (2013- 2023). Int J Health Policy Manag. 2023;12:8124. doi:10.34172/ijhpm.2023.8124

Supplementary file 1. IJHPM Editors Including in-house Editors, Editorial Board Members, and Staff Members.

Table S1. IJHPM Editors

	Name	Affiliation	Country	Continent	Joined IJHPM	Role/s
	In-house editors					
1	Akram Khayatzadeh-Mahani	University of Regina	Canada	North America	January 2013	Founding editor (June 2013-current)<br>Editor-in-chief (June 2013-September 2021)
2	Ali-Akbar Haghdoost	Kerman University of Medical Sciences	Iran	Asia	June 2013	Editor-in-chief (October 2021-current)<br>Director-in-charge (June 2013-September 2021)
3	Mohammad Karamouzian	University of Toronto	Canada	North America	August 2021	Associate editor
4	Vahid Yazdi-Feyzabadi	Kerman University of Medical Sciences	Iran	Asia	August 2021	Associate editor
5	Mohammad Reza Baneshi	University of Queensland	Australia	Oceania	September 2021	Associate editor
	Editorial board members					
1	Marianna Fotaki	University of Warwick	United Kingdom	Europe	June 2013	Editorial board member
2	Russell Mannion	University of Birmingham	United Kingdom	Europe	June 2013	Editorial board member
3	Naoki Ikegami	Keio University	Japan	Asia	June 2013	Editorial board member
4	Ruairí Brugha	Royal College of Surgeons in Ireland	Ireland	Europe	June 2013	Editorial board member
5	Amirhossein Takian	Tehran University of Medical Sciences	Iran	Asia	June 2013	Editorial board member
6	Gill Harvey	Flinders University	Australia	Oceania	June 2013	Editorial board member
7	Jo Rycroft-Malone	Lancaster University	United Kingdom	Europe	June 2013	Editorial board member
8	Aidin Aryankhesal	Iran University of Medical Sciences	Iran	Asia	June 2013	Editorial board member
9	Jeremy Shiffman	Johns Hopkins University	United States	North America	June 2013	Editorial board member
10	Sharon Friel	The Australian National University	Australia	Oceania	June 2013	Editorial board member
11	Nir Eyal	The State University of New Jersey	United States	North America	June 2013	Editorial board member
12	Ole Frithjof Norheim	University of Bergen	Norway	Europe	June 2013	Editorial board member
13	Maria Goddard	University of York	United Kingdom	Europe	June 2013	Editorial board member
14	Rob Baltussen	Radboud University Medical Center	The Netherlands	Europe	June 2013	Editorial board member
15	Mohammad Hajizadeh	Dalhousie University	Canada	North America	June 2013	Editorial board member
16	Elizabeth Bradley	Vassar College	United States	North America	May 2014	Editorial board member

	Name	Affiliation	Country	Continent	Joined IJHPM	Role/s
17	Ilona Kickbusch	Graduate Institute for International and Development Studies	Switzerland	Europe	July 2015	Editorial board member
18	Martin McKee	London School of Hygiene and Tropical Medicine	United Kingdom	Europe	July 2015	Editorial board member
19	Victor G. Rodwin	New York University	United States	North America	September 2015	Editorial board member
20	Ronald Labonté	University of Ottawa	Canada	North America	September 2015	Editorial board member
21	Agnes Binagwaho	University of Global Health Equity	Rwanda	Africa	November 2016	Editorial board member
22	Lawrence O. Gostin	Georgetown University Law Center	United Kingdom	Europe	November 2016	Editorial board member
23	Viroj Tangcharoensathien	Ministry of Public Health	Thailand	Asia	November 2016	Editorial board member
24	Eivind Engebretsen	University of Oslo	Norway	Europe	August 2022	Editorial board member
	Staff members					
1	Hafez Hassanzadeh	Tabriz University of Medical Sciences	Iran	Asia	June 2013	Production manager
2	Sahar Najafizadeh	Kerman University of Medical Sciences	Iran	Asia	May 2013	Managing Editor (January 2017-current)<br>Technical Editor (September 2013-December 2016)<br>Assistant Editor (May 2013-August 2013)
3	Mina Moradzadeh	Kerman University of Medical Sciences	Iran	Asia	August 2013	Managing editor
4	Reza Khosravi	Tabriz University of Medical Sciences	Iran	Asia	June 2013	Consultant
5	Fatemeh Yazdizadeh	Kerman University of Medical Sciences	Iran	Asia	February 2017	Assistant editor
6	Fatemeh Molaee	Kerman University of Medical Sciences	Iran	Asia	November 2018	Assistant editor
7	Zeynab Golestanikhah	Kerman University of Medical Sciences	Iran	Asia	May 2022	Assistant editor
