# Supplementary material for: International Journal of Health Policy and Management (IJHPM): A Decade of Advancing Knowledge and Influencing Global Health Policy (2013-2023)
Source: Int J Health Policy Manag. 2023 May 24;12:8124. doi: 10.34172/ijhpm.2023.8124 (PMC10425691; doi:10.34172/ijhpm.2023.8124)
Supplement: Supplementary file 2 — IJHPM International Seminar’s Agenda. [file ijhpm-12-8124-s002.pdf]

1 DAY INTERNATIONAL SEMINAR

Improving Quality and Efficiency of Health Services by Introducing Market Incentives, Competition and Choice

April 19, 2014

Venue:
Conference Hall, Pars Hotel
Kerman

Organizer:
International Journal of
Health Policy
and Management (IJHPM)
Institute for Futures Studies
in Health
Kerman University of
Medical Sciences
Kerman, Iran

OPPORTUNITIES & CHALLENGES

Session One International Lessons

Welcome and Introduction	8:30-9:00	Professor Ali-Akbar Haghdoost<br>Dr. Akram Khayatzadeh-Mahani<br>Kerman University of Medical Sciences (Iran)
Improving Quality and Efficiency of Health Services by Introducing Market Incentives, Competition, and Choice: Opportunities and Challenges	9:00-9:45	Professor Marianna Fotaki<br>Warwick University (UK)
Discussion on Marianna Presentation	9:45-10:00	All Participants

Break
10:00-10:30

Markets, Competition and Choice: An American Perspective	10:30-10:50	Professor Carol Molinari<br>Baltimore University (USA)
Markets in the Public Sector, Choice or Voice, and Decentralizing Health Services; Lessons from NHS	10:50-11:10	Professor Ian Greener<br>Durham University (UK)
Markets, Competition and Choice: Lessons for Canada	11:10-11:30	Dr. Owen Adams<br>Canadian Medical Association (Canada)
Why is Public Health Care Always Best?	11:30-12:00	Dr. Alex Scott-Samuel<br>Liverpool University (UK)

Wrap Up and Summary of International Lessons	12:00-12:30	Professor Marianna Fotaki

Lunch

12:30-14:00

Session Two National Voices

Improving Quality and Efficiency of Health Services in Iran: Opportunities and Challenges	14:00-14:30	Dr. Ali Akbari-Sari<br>Tehran University of Medical Sciences (Iran)
Family Physician: The Second Biggest Reform in the Iranian Health System	14:30-15:00	Dr. AmirHosein Takian<br>Brunel University (UK)
Closing Address	15:00-15:30	Professor Marianna Fotaki

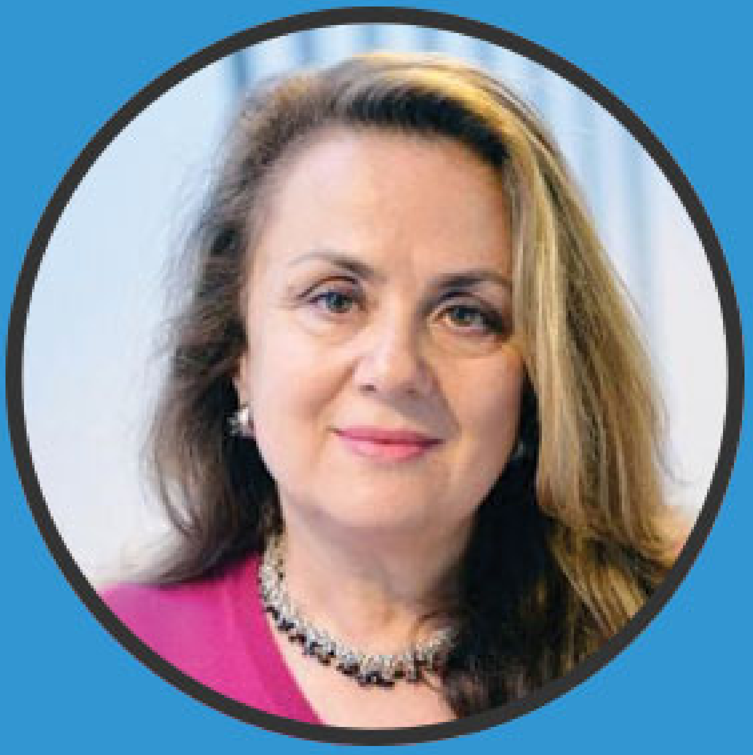

Professor MARIANNA FOTAKI

Warwick Business School, Warwick University, UK

Biography:

Marianna Fotaki is Professor of Business Ethics at Warwick Business School and a visiting professor at The University of Manchester. Before joining academia Marianna has worked as EU resident adviser to the governments in transition and as a medical doctor for Médecins Sans Frontières and Médecins Du Monde for ten years in total. She is a graduate of medicine, public health, and has obtained a PhD in public policy from the London School of Economics and Political Science.

Research Interests

Public And Health Policy Management:

With a focus on: (i) market reforms in health care in EU countries and economies in transition and (ii) the impact of patient choice and competition on promoting new forms of public private partnerships and developing a new category of users/consumers. Her work in this area connects conventional approaches to understanding policy making with new theoretical perspectives drawn from critical social theory such as psychosocial studies.

People in Organizations:

With a particular focus on: (i) user-provider interface and leadership in public services and (ii) measures to counteract exclusion and promote diversity in organisations. Her research in this area applies feminist theory and intersectionality perspectives to developing deeper understandings of gendered forms of organization and counteracting exclusion in organizations and society.

The Impact of Business in Society:

Including (i) ethics of diversity (ii) ethics of care and consumerism and (iii) social enterprise and public-private partnerships. She is also particularly interested in the role of business education in promoting fairness in society.

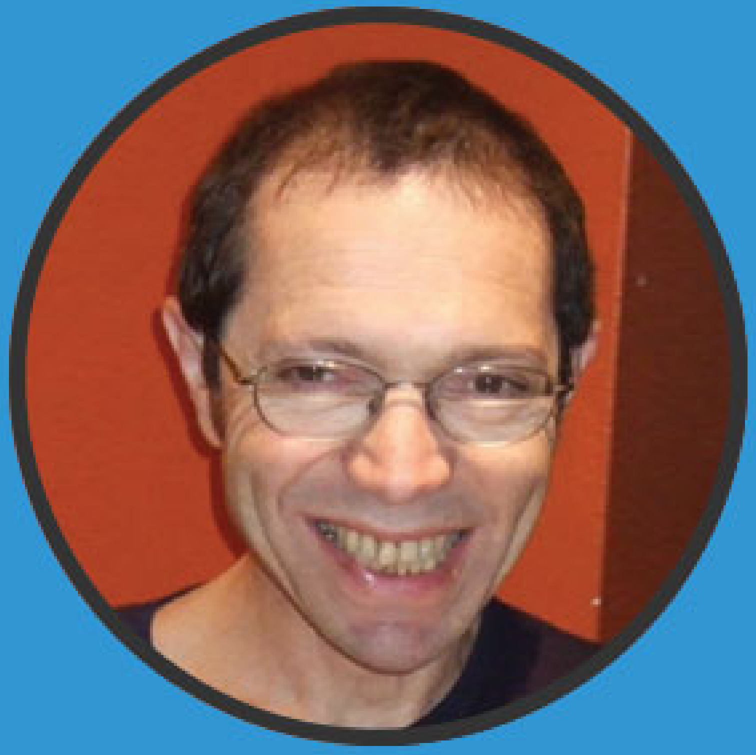

Doctor ALEX SCOTT-SAMUEL

Health Inequalities and the Social Determinants of Health Department, University of Liverpool, UK

Biography:

Alex qualified in medicine at the University of Liverpool in 1971, and took his Master in Community Health in 1976. From 1978-94 he was consultant in Public Health with Liverpool Health Authority. From 1979-85 he was founding editor of the journal Radical Community Medicine (now Critical Public Health). Together with Peter Draper, he established the Public Health Alliance (now the UK Public Health Association) in 1986. In 2003, he was a co-founder of the Politics of Health Group. He is Vice Chair of the Pioneer Health Foundation (founders of the Peckham Experiment).

Research Interests

Most of his health-related interests are linked with politics and public policy. He has worked with health inequalities since the late 1970s; his 20 years in the NHS have enormously influenced his academic career.

Academic Position

Since 1994, Alex has been Senior Clinical Lecturer in the Division of Public Health at the University of Liverpool, where he directs IMPACT (the International Health Impact Assessment Consortium); Liverpool Public Health Observatory and EQUAL (the Equity in Health Research and Development Unit).

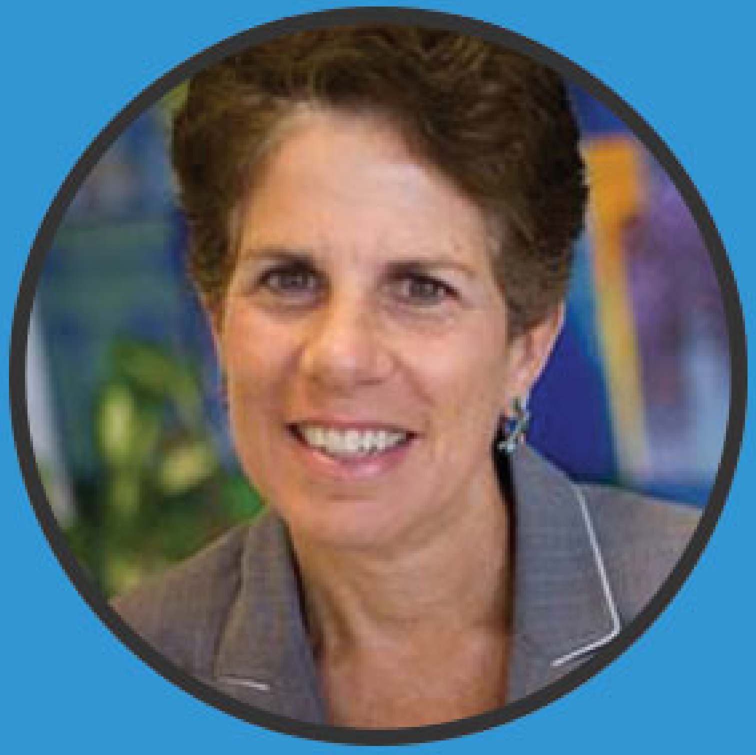

Professor
CAROL MOLINARI

School of Health and Human Services, Baltimore University, USA

Biography:

Carol Molinari is an Associate Professor of Health Systems Management in the College of Public Affairs. Carol has taught undergraduate and graduate students in universities across USA; the most recent was at Jefferson College of Health Sciences and Virginia Tech Carilion Medical School in Roanoke, VA.

Research Interests:

Carol research has focused on ways that leadership and governance can make a difference in the performance of healthcare organizations. Her current research focuses on studying culturally competent ways for healthcare organizations to lead and manage diverse clients and workers. In particular, she is studying ways that long term care facilities are managing and delivering care to increasingly diverse residents and patients. Another current strand of her scholarship involves examining effective pedagogy for online and hybrid/blended courses. She has presented and continues to present and publish in various academic healthcare management journals.

Executive Positions:

Professor Molinari has served on governing board of an integrated health system in Washington State and has also provided board service on several not-for-profit community boards. She has been a program director for several Healthcare Management programs and has been an executive director of a technology council in Spokane, WA.

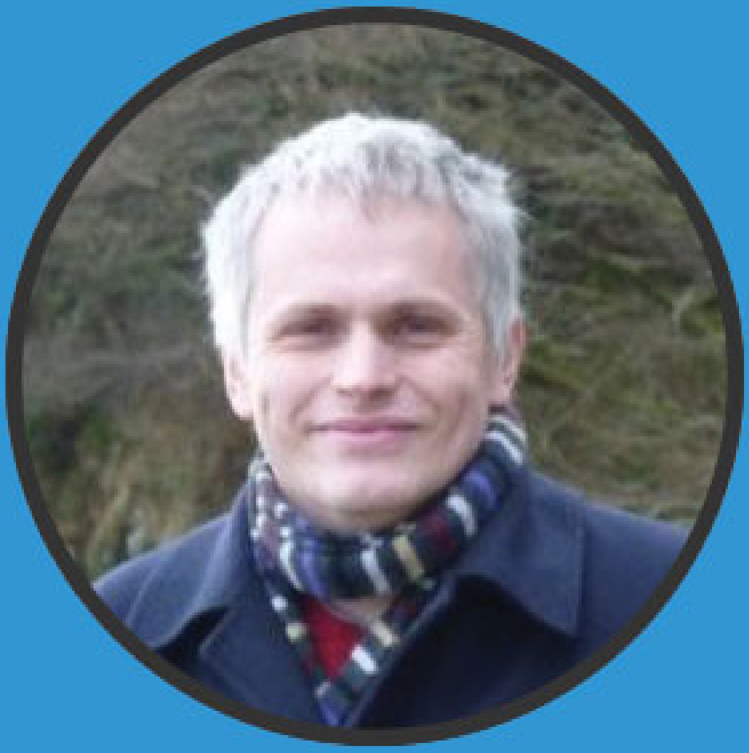

Professor IAN GREENER

School of Applied Social Sciences, Durham University, UK

Biography:

Ian Greener is a Professor in the School of Applied Social Sciences at Durham University where he leads the provision of research methods teaching on ESRC-accredited courses. Prior to this he worked for a number of UK Higher Education Universities since 1997 including Manchester and York in Business Schools and Social Science Departments. Before that he worked as an accountant in the He has published in a wide range of journals in social policy, sociology, political science and organisation studies..

Research Interests:

Social policy and public management, with a particular concern with healthcare and health policy

Academic and Executive Positions

He is a member of the Department of Health Service and Delivery Research commissioning Board, as well as commissioning research on their RiSe research programme. He is currently a member of the SPA Executive, and on the editorial Board of the journal Social Policy and Administration.

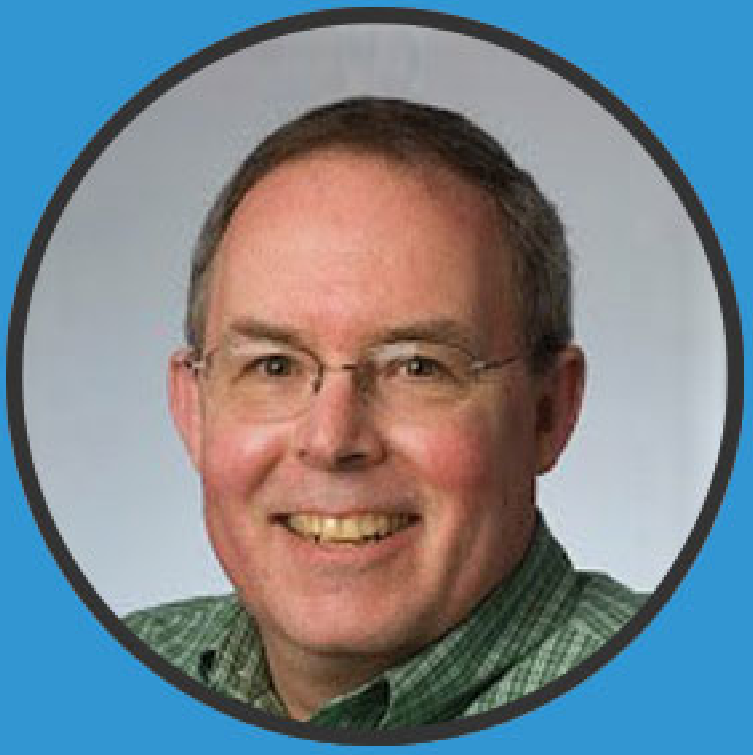

Doctor OWEN ADAMS

Canadian Medical Association, Ottawa, Ontario, Canada

Biography:

Owen Adams is the Assistant Secretary General, of Research, Policy and Ethics at the Canadian Medical Association (CMA). The Research, Policy and Ethics Directorate is a multidisciplinary team that carries out a variety of policy research projects in the areas of health system financing and reform, medical economics, medical ethics and medical workforce issues. Prior to joining the CMA in 1990, Owen was a senior research analyst in the Health Statistics Division of Statistics Canada, which he joined in 1978. He holds a BA and MA in Sociology from the University of Western Ontario (London, ON).

Academic and Executive Positions:

Throughout the 1980s, Owen taught graduate courses on a part-time basis in quantitative research methods and social aspects of health care in the Master of Health Administration Programme at the University of Ottawa. He is presently an Adjunct Assistant Professor in the Department of Community Health and Epidemiology at Queen's University. He has written in the field of population health status indicators, and has collaborated extensively in the analysis and publication of vital statistics and population-based health survey data. At the CMA Owen collaborated on a number of reports in the area of physician resource planning and the evolving Canadian health care system.

Along with serving as a director on the Care for Corporate Board, Owen is the Chair of the Fundraising & Allocations Committee as well as the Nominations Committee, and serves on the Finance, HR & Risk Management Committee as well as the Ottawa Program Board.
